# The Social Evil

**Published:** 1911-04

**Authors:** Winslow Anderson


### "The Social Evil"

BY WINSLOW ANDERSON, M. D.

The social evil, with its attendant crimes and diseases, has engaged the earnest attention of the ablest medical men, sociologists, hygienists, venereologists and statesmen of every country from the times of Abraham and Moses. At one time prostitution was punishable by placing the unchaste woman in an iron cage and dipping her in the river until she was almost drowned. At another time the impure woman had her nose cut off or she was branded with a red hot iron. The fallen woman has been publicly whipped. She has been obliged to wear distinguishing dress. At the time of Christ she was stoned to death. During the crusades and later to prevent a possible breach of observing chastity, the fair ones had their labia minora *padlocked.* In other cases steel girdles—"girdles of chastity"—were fastened about the waist and limbs in such a manner as to guard the vulval orifice. Napoleon, one hundred years ago, was of the opinion that cohabitation was a "physical necessity" for his soldiers, but he did not wish to minimize their fighting strength by having them exposed to unhygienic surroundings, so he devised a system of "regulation" with medical inspection. Since his time Scandinavia provided supervision over the gaities of life with compulsory notification of venereal diseases. Neisser, who discovered the diplococcus or micrococcus of gonorrhea in 1879, has given Germany a highly complicated restrictive registration and medical inspection system. In 1896 Berlin had about 4000 registered, licensed, inspected abandoned women, with over 50,000 clandestine or unregistered lewd women. Paris in 1903 under *Police des Moeurs* (Morals police) had registered some 5000 women that legally carried on commercial vice. They were inspected by medical men and specially policed according to law. During the same period there were over 50,000 other immoral women on the streets that were neither registered nor examined. During the past thirty years Paris registered and inspected 150,000 licensed demi-mondes and arrested 725,000 clandestines that were found carrying on indiscriminate sexual intercourse without a license. In these "arrests" many innocent women were branded "immoral" on account of "mistakes" or malice or for purposes of blackmail.

The continental system of regulating vice has been abandoned generally excepting in Geneva, where houses of "ill-fame" are "tolerated" or licensed. Great Britain about forty years ago enacted regulatory registration for "fallen women." Police in plain clothes—spies—were empowered to arrest any woman they had "reason to believe" immoral. The policeman's "declaration" was sufficient to blast the reputation of any woman. These arrested women were requested to sign a paper called "voluntary submission." Many were ignorant and easily coerced into signing this "admission." If these women, innocent or abandoned, refused a medical examination they were sent to prison for disobedience. If found diseased they were sent to a hospital prison. If resistant they were imprisoned for months with hard manual labor. Under this regime the police spies held the power of intimidating or arresting any "suspect." They often acted on "hints from outsiders," or for reasons of jealousy or revenge, or for purposes of blackmail and extortion. The whole object of this "law of regulation" has failed. In 1871 a Royal Commission recommended the abolition of compulsory examination. It took twelve years for this to become law. The legal age of protection for girls was twelve years! Twelve years later this was raised to thirteen years—it is now sixteen years. England has now no "legalized," registered houses of commercialized vice. One English journal states recently that the reason for an outbreak of syphilis in an African province was "the introduction of Christianity!"

Norway abolished *Morales Police* in 1888.

Italy has also abolished "regulations."

France no longer punishes sexual irregularity.

St. Louis tried to "regulate" prostitution from 1870 to 1874 and abolished it.

New Orleans has her denizens of the "red light district" legalized, licensed and under police surveillance still.

In the Japanese Empire a large city—near Tokyo—which contains some 8000 inmates, mostly young girls of tender years, is specially devoted to legalized or criminal vice. Ten, twenty or fifty maidens in each house, are poised—like dolls in their show windows—for sale.

Neisser of Germany advocates the recognition and tolerance of indiscriminate lewdness as a trade, unavoidable under present social conditions. He advocates teaching the principles of chastity to young men. Neisser also recommends the examination of every man who enters a house devoted to licentiousness.

Civilized countries for the past half century have tried all known methods of moral and legal prophylaxis. At the various world's congresses, medical men, scientists, sociologists and moralists, ably assisted by the clergy, have advocated regulations, inscription sequestration, medical inspection, police surveillance, up to and including licensing or *legalizing* a life of shame, in an endeavor to regulate sexual irregularity.

A short time ago a noted member of the German Reichstag said: "The State which officially tolerates and guarantees houses of prostitution assumes the rôle of procurer."

M. Jules Faure said lately: "The worst that could befall the public health is nothing to the corruption of morals and national life engendered, propagated and prolonged by the system of official surveillance."

THE WHITE SLAVE TRAFFIC.

At the present time the consensus of opinion held by the vast majority of moralists is that all "systems" of regulation, registration, isolation, compulsatory medical inspection, civil espionage, and licensing of abandoned women, *legalizes* promiscuous sex-immorality. It *increases* the notorious traffic in young girls for immoral purposes and *actually increases vice and disease.* Licensing or legalizing moral obliquity has so far increased the dangers of contamination.

Statistics show that 50 per cent of minor girls are affected with venereal diseases. Twenty-five per cent of them have contracted syphilis. Licensing commercialized vice increases clandestine or illicit sex immorality. Of this latter class of strumpets 72 per cent have been found to be diseased. Seventy-five per cent of all prostitutes begin before the age of twenty-one.

It is claimed that the great wealth of Corinth proceeded largely from the foul hire of prostitution in the temples.

*The Gonococcus.*—Eighty per cent of blindness at birth is caused by this infection. Twenty-five per cent of all blindness is caused by the same disease. Fifty per cent of sterility in women and twenty-five per cent of sterility in men is from gonorrhea.

In the United States there are probably no less than 500,000 women in houses of "ill-fame," and twice as many clandestines outside of them. It is estimated that 450,000 young men in America contract venereal diseases each year.

It is stated on the best authority that 65,000 American girls and 15,000 foreigners are being trapped and sold yearly in the United States into houses of defamation. Illegal marriages entrap many, promise of marriage, of employment, deceptive invitations, decoy others. Young girls are stolen and carried off by force. The stings of poverty and destitution, betrayals and enticements under various pretexts fill the "licensed" brothels in the larger cities.

Dr. W. F. Radue, in *Clinical Medicine* for January, writes:

To go into the red-light districts of our great cities and drive out the denizens is folly, for the certain result will be the same as it was in New York some years ago. When these prostitutes were driven from their quarters, from place to place, they finally drifted into the apartment and tenement houses and the respectable residence districts, there coming in contact with pure, innocent girls, poisoning their minds, and frequently leading them on to share their own miserable fate in a life of shame, and, as they call it, of "easy money."

That we have societies and private individuals who are doing good and are trying to reform some of these people I admit, but to do any appreciable good we must go at them with greater vigor and by different methods, varied as each case may demand. While some are willing to be helped, many of these unfortunates do not and will not change their mode of life—the life of the prostitute—for something better and purer.

For the latter kind something else must be done. To try to reform those that will not be reformed is a waste of time and money, so that the only thing left is to restrict them to a circumscribed part of the town where they can be found by those who so desire, making it an offense punishable by fine and imprisonment if they are found outside their limits. This must be made a State law. Moreover, this whole matter should be taken out of the hands of the police department and the low politicians who for years have been bleeding these unfortunates to the extent of unnumbered thousands of dollars, enriching themselves with this blood-money.

These women should be under the supervision of the city physician and should be examined at frequent intervals. This to avoid the spread of venereal diseases as much as possible. To condemn these people is not of the spirit of that great reformer Jesus Christ, who said, "Let him who is without sin among you cast the first stone." To stop prostitution is utterly impossible. You can not stop it any more than you can stop the sun from shining—no power on earth can stop it. But it is the duty of every honest man and woman to try to get this evil regulated so that these women may not go about contaminating the innocent, while they also should work for the improvement of all places where the still pure may be tempted, such as offices, factories, department stores, and all places where men and women work side by side. That thousands of girls are brought to their downfall in such places and under such conditions is a positive fact. Any one who could bring about a betterment of this condition would be immortalized.

One thing is certain, prostitution among our women will not down by force. It must be overcome by kindness and by the extension of a willing and helping hand, aiding them in all possible ways to overcome this passion for evil for one of goodness and purity. And if our men of wealth would only spend some of their money in this direction, it would be more glorious for them than to build churches that too often invite only the rich while ostracizing the lowly and lonely.

What we need is men and women of broad and liberal minds, who are disgusted with the condition of things as they are, those who will get out and work royally and unselfishly for the upliftment of these women and try to save those that are willing to be saved from destruction and untimely death, bringing them to realize what they are doing and the dire results their mode of life will bring them. Those who are willing to put their hearts into this work and can learn to do it well will some time be immortalized.

That this subject is not new I am well aware, but as this condition of things is getting the upper hand it must be fought vigorously, and brought to the notice of fathers and mothers, and the public above all; and our Legislatures must compel the latter to do something for the abatement of this most degrading and soul-destroying vice—the social evil.

(In the main we agree with Dr. Radue. The subject which he discusses is one of vital interest, and there can be little question that there is an increase of moral laxity, both among men and women. The problem is not one that concerns either sex alone, and in our opinion it is not one that can be solved by segregating the young women from the young men, as he so warmly advocates. The association of the two sexes is perfectly natural in the home, in social and business intercourse, in the church, and in most schools. To set one sex apart from the other, in defiance of the laws of nature and in quasi-ignorance, is but to put a premium upon the secret relationship which too easily becomes illicit.—Ed. of *Clinical Medicine.*)

The editor of *Clinical Medicine* says:

Candidly, I do not believe that we are ever going to crush out the sexual diseases by enacting laws which wreak the vengeance of society upon the sufferer. We must tell the truth about these diseases, tell it to young women and young men, so that, if they err, they will do so with their eyes open. Ignorance and poverty are probably more important factors in the propagation of syphilis and gonorrhea than passion—though this must be reckoned with. * * *

NOTED CLUB WOMAN FAVORS POLYGAMY AS A CURE FOR THE "SOCIAL EVIL" AND DIVORCE.

"Polygamy is the most feasible of all the panaceas that have been put forward for the cure of the divorce evil.

"I favor polygamy for those who want it."

It is not an elder in the Mormon church who is speaking; nor the shade of Brigham Young, or even one of the forty-three childred of Joseph Smith.

Mrs. Mortimer M. Menken, wife of a prominent Metropolitan attorney, is the one woman in a city full of suffragettes and "modern women" who dares to raise her voice in defense of the plural marriage system.

Mrs. Menken, who is a member of the Equal Franchise Society, the Political Study Society and the Parliament, attended the mock meeting of the women Governors of the latter organization at the Waldorf-Astoria. Each of the women present represented a State. Mrs. Menken was the Governor of Utah, and she threw the audience into a panic when she boldly declared herself in favor of a return to the practice of polgyamy.

In speaking to a reporter Mrs. Menken laughed heartily when asked if she favored the adoption of polygamy for all women.

"My beliefs are hardly as drastic as that," she replied. "Now, I did not say that I should advise all men to get themselves half a dozen wives, or women to band themselves into clubs of a dozen or more members and marry some man.

"But from my first-hand observations in Utah I believe polygamy makes for morality. It is even spreading into the surrounding States, Idaho in particular.

"The people are beginning to realize the many benefits to be derived from polygamy. Polygamy, for instance, settles the labor question. In Utah you see the husband and his four, six or eight wives, as the case may be, tilling the soil in perfect harmony. All take a personal interest in the farm, and the result is that the family is independent."—*Ex.*

PREVENTION OF PROSTITUTION.

In one hundred years of licensing, inscribing and inspecting, commercial abandonment has grown apace. It has existed from the beginning of time, and if we are to minimize sexual immorality new methods must be inaugurated. Police surveillance, arrests, segregation and licensing have made poor progress. Disease contamination is increasing. In most "civilized" countries from 50 per cent to 80 per cent of the men are infected. Prostitution is a moral disease depending upon *ignorance, poverty* and *want.* Only a small proportion depends upon moral degeneration, passion and lust.

To preserve the "mystic key to Christian holiness," we must educate the *young men* as well as the young women. Instruction should be given in our schools. The physiology and hygiene of the sexual organs should be taught the *boy* as well as girl. The phenomena of the reproductive and generative organs should be taught without *prudery, hypocrisy* or *sentimentality.* The dangers of contamination by the gonococcus, the spirochaeta pallida and the Ducrey-Unna bacillus should be early instilled into the minds of the young. Mothers and fathers should be instructed, that they may teach their children. *Education* will do more to minimize vice and disease than any other force ever employed.—*Pacific Medical Journal.*

